# Intensive Care Unit-Acquired Weakness: A Review of Recent Progress With a Look Toward the Future

**DOI:** 10.3389/fmed.2020.559789

**Published:** 2020-11-23

**Authors:** Wenkang Wang, Chuanjie Xu, Xinglong Ma, Xiaoming Zhang, Peng Xie

**Affiliations:** ^1^Department of Critical Care Medicine of the Third Affiliated Hospital (The First People's Hospital of Zunyi), Zunyi Medical University, Zunyi, China; ^2^Department of Molecular and Cellular Biology, Baylor College of Medicine Houston, Houston, TX, United States

**Keywords:** intensive care unit-acquired weakness, pathogenesis, diagnosis, treatment, neuromuscular junction

## Abstract

Intensive care unit-acquired weakness (ICU-AW), a common neuromuscular complication associated with patients in the ICU, is a type of skeletal muscle dysfunction that commonly occurs following sepsis, mobility restriction, hyperglycemia, and the use of glucocorticoids or neuromuscular blocking agents. ICU-AW can lead to delayed withdrawal of mechanical ventilation and extended hospitalization. Patients often have poor prognosis, limited mobility, and severely affected quality of life. Currently, its pathogenesis is uncertain, with unavailability of specific drugs or targeted therapies. ICU-AW has gained attention in recent years. This manuscript reviews the current research status of the epidemiology, pathogenesis, diagnosis, and treatment methods for ICU-AW and speculates the novel perspectives for future research.

## Introduction

Around 13–20 million people worldwide receive treatment in intensive care units (ICUs) annually (1). Data from medical institutions worldwide show the incidence of ICU-acquired weakness (ICU-AW) ranging from 25 to 31% (2–5), with ~3.25–6.2 million new patients annually. The incidence of ICU-AW varies depending on the age, sex, primary diseases, and treatment. Up to 70% of elderly ICU patients may have complications with muscle atrophy. As the skeletal muscle is correlated with immune function and glucose and protein metabolism, such patients have significantly higher mortality (6). In surgical ICUs, 56–74% of patients show symptoms of ICU-AW (7). When combined with sepsis; hyperglycemia; bedridden status; long-term mechanical ventilation; and use of glucocorticoids (GCs), neuromuscular blocking agents (NMBAs), and vasoactive drugs, the risk of ICU-AW can increase (8).

Patients with ICU-AW may have critical illness polyneuropathy (CIP) and critical illness myopathy (CIM). With the progress in ICU-AW research, molecular mechanisms, such as local immune activation triggered by cytokines and microcirculation defects, were found to be accompanied by both myogenic and neurogenic damages, increasing the difficulty of determining the primary pathogenesis (9). Thus, the classical dichotomy was gradually abandoned. Currently, critical illness polyneuromyopathy (CIPNM) is a more versatile concept widely used to demonstrate the multilevel and multifactorial pathophysiological basis of the disease. ICU-AW is characterized by symmetrical limb weakness, with more severely affected proximal limb muscles in the shoulders and hips. Moreover, respiratory muscles are often affected, especially in patients receiving mechanical ventilation, and is associated with difficulty in weaning from the ventilator and atrophy (10). However, this phenomenon can also occur in ventilator-induced diaphragmatic dysfunction (VIDD) and should be distinguished from ICU-AW. In VIDD, the phrenic nerve signal transmission and signal transduction at the neuromuscular end-plate appear normal, thus distinguishing it from polyneuropathic forms of ICU-AW (11), which show conduction abnormalities in electrophysiological studies. The clinical complications of ICU-AW include difficulty withdrawing the ventilation machine, paresis or quadriplegia, decreased reflexes, and muscle atrophy. ICU-AW has a long disease course that can occur beyond hospitalization, persisting for several years after discharge (12). ICU-AW is independently associated with higher post-ICU mortality and clinically relevant lower physical functioning in survivors at 6 months after ICU discharge (13). Additionally, ICU-acquired neuromuscular complications may impact 5-year morbidity and mortality (14).

Currently, despite limited clinical evaluation standards for ICU-AW, the Medical Research Council score (MRC score) is widely used for its evaluation and diagnosis (15, 16). However, the MRC score has major limitations requiring patients to be sufficiently awake and cooperate. Recent studies have shown that for uncooperative patients, ultrasound and evaluation of twitch force after magnetic nerve stimulation could be used as an alternative to the MRC scale to assess the degeneration of muscle function and development of ICU-AW (17, 18). Besides, electromyography (EMG) is another choice that reduces the subjective errors of healthcare professionals, and distinguishes between CIP and CIM, the main differential diagnoses in ICU-AW. However, EMG is not routinely used in clinical practice because it is time-consuming and costly. In fact, MRC and EMG are complementary in the clinical setting. Finally, nerve or muscle tissue biopsy is rarely performed in clinical practice.

Currently, treatments for ICU-AW mostly depend on nutrition and supportive therapies to relieve symptoms as specific drugs and treatments are lacking, which is challenging for ICU clinicians. Therefore, exploring the pathophysiological mechanism of ICU-AW and seeking specific therapeutic drugs and strategies are critical for investigation.

## Common Risk Factors

### Multiple Organ Failure

Multiple organ failure (MOF), a clinical syndrome with simultaneous or sequential failure of two or more organs following severe infection, trauma, major surgery, or pathological obstetric complications, is one of the most common risk factors of ICU-AW. When patients are in the MOF state for a long duration, ICU-AW may occur. The primary causes of MOF are sepsis and septic shock, and more than 70% of patients with sepsis were reported to develop ICU-AW (11).

Sepsis and MOF lead to mitochondrial dysfunction, decreased respiratory chain complex I activity, decreased adenosine triphosphate levels on skeletal muscle biopsy (19), and excessive free radicals in tissues, causing muscle atrophy. Although sepsis was considered the key risk factor for ICU-AW in the past, patients with acute pancreatitis, multiple injuries, and cardiac arrest without sepsis also developed MOF leading to ICU-AW (20). Hence, sepsis is not an independent risk factor for ICU-AW.

### Mobility Restriction

When patients are bedridden for a long duration, various stimuli are reduced with declined systemic or local physiological functions. Furthermore, symptoms such as joint contracture, pulmonary infection, bedsores, deep vein thrombosis, constipation, and muscle atrophy may occur. In the disuse state, the muscle mass and volume decrease, the muscle fiber cross-section area shrinks, and the muscle fiber type changes from type I to type II, with the degree of muscle atrophy correlated with the duration of disuse state and the patient's age (21).

In addition to structural changes, muscle strength also decreases significantly. When a healthy adult is bedridden, the muscle strength is reduced by 1% per day. Long-term muscle inactivity causes changes in mitochondrial function, leading to an increase in reactive oxygen species (ROS), inducing muscle atrophy and dysfunction (22). Early rehabilitation may prevent ICU-AW and shorten the mechanical ventilation duration, with appropriate resistance exercise restoring the quantity and strength of muscles (23).

### Hyperglycemia

Hyperglycemia refers to a blood glucose level higher than the normal value (3.9–6.1 mmol/L). Although glucose is a highly effective nutrient for critically ill patients, excessive accumulation may occur under stress, causing exaggerated inflammatory responses, decreased complement activities, immune system imbalance, and mitochondrial damage (24). Since fructose kinase is not expressed in nervous tissue, accumulation of large amounts of sorbitol and fructose may occur in the nerve cells, resulting in an intracellular hyperosmotic state causing nerve cell swelling, degeneration, and necrosis.

Recent studies have shown that hyperglycemia affects the respiratory muscle functions, leading to ICU-acquired respiratory muscle weakness and adverse prognoses increasing patient mortality (25). Van den Berghe et al. found that intensified insulin therapy in ICU patients (i.e., administering insulin to control the blood glucose levels between 80 and 110 mg/dL) significantly reduced the duration of mechanical ventilation and hospital stay (26). ICU patients complicated with diabetes or hyperglycemia may develop peripheral neuropathies due to metabolic disorders, oxidative stress, neurotrophic factor deficiencies, or vascular injuries (27).

### Glucocorticoids

GCs not only regulate the biosynthesis and metabolism of carbohydrates, lipids, and proteins, but also have strong anti-inflammatory and anti-fibrotic effects, rapidly treating septic shock. Long-term use of low to moderate doses of GC in acute respiratory distress syndrome (ARDS) can reduce inflammatory cytokine transcription, mitigate systemic and pulmonary inflammation, improve hypoxemia, and reduce the mechanical ventilation time and mortality. Therefore, GCs are the main choice for treating certain primary diseases in critically ill patients.

However, GCs have a direct catabolic effect on skeletal muscles, with their long-term use causing type II muscle fiber atrophy and proximal muscle weakness (28). Keh et al. showed that 34% of patients with sepsis and 45% of non-septic patients developed ICU-AW after GC usage (29). ICU-AW caused by excessive GC use is difficult to diagnose due to insignificant changes in muscle enzymes and lack of specificity and diversity in the electrophysiological results. Currently, the incidence of the disease related to GC use can only be determined with clinical symptoms (30). The key to GC administration is to reduce the dosage and frequency. Exercise may alleviate GC-induced muscle atrophy, but no specific treatment is available (31).

### Neuromuscular Blocking Agents

Depending on their mechanisms of action, NMBAs can be classified as depolarized and non-depolarized. Although the molecular structures of a depolarized muscle relaxant (succinylcholine) and acetylcholine are similar, the effect lasts longer. In contrast, a non-depolarized drug (atracurium) competes with acetylcholine for neuromuscular junction (NMJ) receptors without activating them, causing skeletal muscle relaxation (32). In the ICU, NMBAs are used for emergency intubation, ARDS, status asthmaticus, elevated intracranial or intra-abdominal pressure, and therapeutic hypothermia after ventricular fibrillation-related cardiac arrest.

Since it was reported that NMBAs could be a risk factor for ICU-AW, its clinical use has reduced. Although NMBAs can improve oxygenation and reduce mortality in moderate to severe ARDS (33), NMBA administration in ICU patients remains controversial. Brunello et al. believed that the correlation of NMBAs with neuromuscular dysfunction (34) and their long-term use increased the risk of muscle atrophy similar to denervation treatment. Conversely, many opponents believe that these results are affected by confounding factors (35) and that NMBA use should be unrestrained. Therefore, further evaluation is needed to determine proper use.

Other factors increase the incidence of ICU-AW, including long-term mechanical ventilation, electrolyte imbalance, hyperosmotic pressure, female gender, hyperproteinemia, aging, parenteral nutrition, inappropriate use of vasoactive drugs (36), high lactate level, and an abnormal calcium ion concentration (20). When studying risk factors, the presence of confounding factors should be considered because ICU patients often have multiple secondary pathophysiological conditions; thus, conducting a “single factor” analysis is important.

## Possible Mechanisms

**Figure 1:**
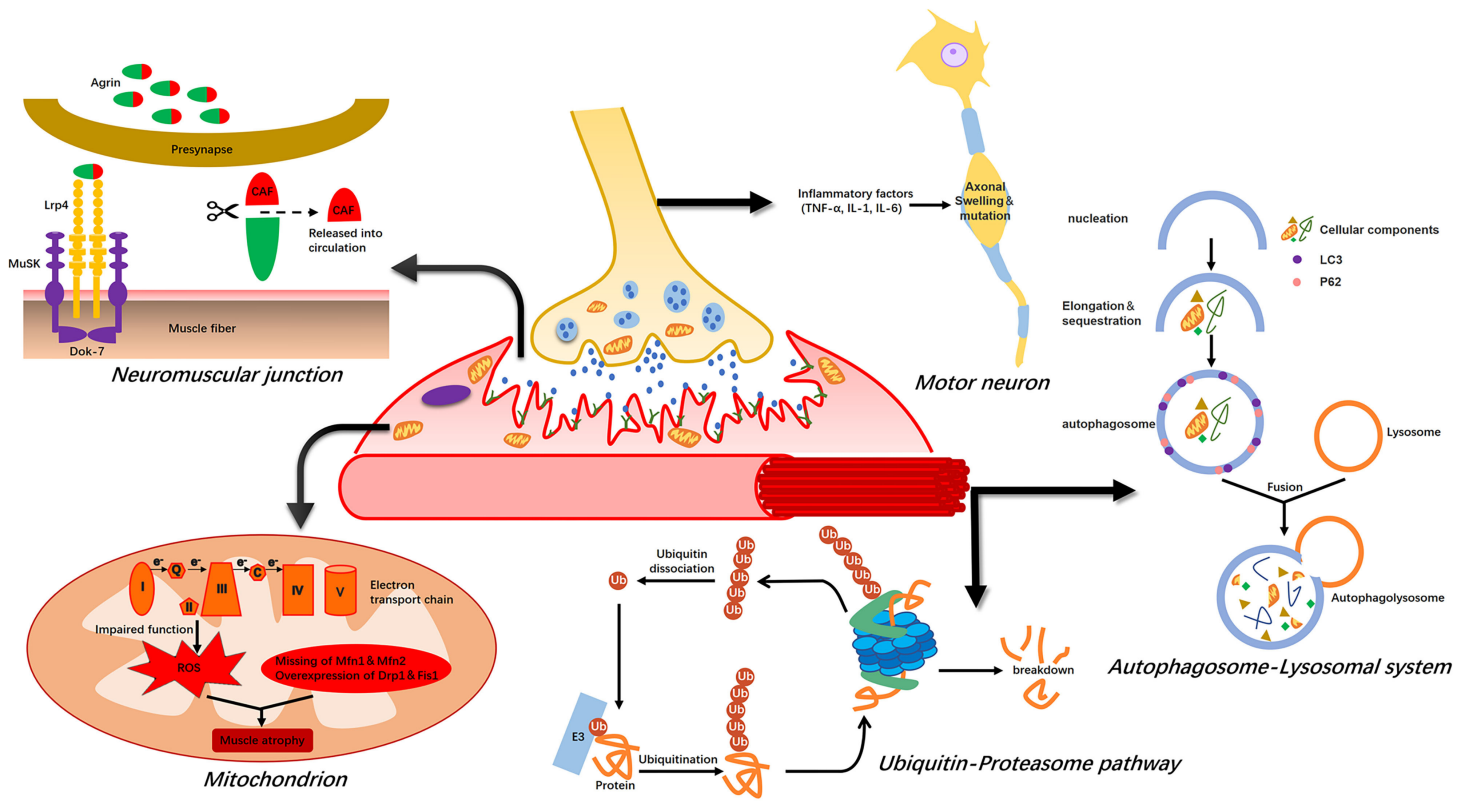
Possible mechanism of intensive care unit-acquired weakness (ICU-AW). Muscle atrophy is caused by an imbalance between protein synthesis and degradation. The ubiquitin–proteasome pathway and autophagy–lysosome system are activated during this period, leading to massive degradation of muscle proteins. ROS released after mitochondrial damage also induce proteolysis. The agrin-MuSK-Lrp4 signaling pathway is impaired during abnormal NMJ function, leading to muscle atrophy. Finally, inflammatory factors can cause axonal swelling in motor neurons, resulting in neurapraxia.

Muscle atrophy occurs due to an imbalance between synthesis and uncontrolled degradation of muscle proteins. In critically ill patients, muscle atrophy is mainly a result of a massive loss of myosin and myoglobin-related proteins in limb and trunk muscles (37). The four major proteolytic systems are the ubiquitin–proteasome system, calpain, caspase 3, and the autophagy–lysosome system. Furthermore, NMJs, mitochondria, and motor neurons may play key roles in ICU-AW pathogenesis (Figure 1).

### Ubiquitin–Proteasome Pathway, Calpain, and Caspase 3

The activity of the ubiquitin–proteasome pathway (UPP), the most important regulator of proteolysis in skeletal muscles (38), increases during the acute phase of muscle atrophy in ICU patients (39). UPP activation or upregulation is induced by several factors, including increased inflammatory factors (TNF-α, IL-1, and IL-6), excessive superoxide and ROS in tissues (40), stress responses, and mobility restriction. Inflammatory cytokines have direct and indirect effects on signaling pathways regulating muscle mass (41) and TNF-α. IL-1 can increase ubiquitin gene transcripts, thereby accelerating skeletal muscle catabolism. Upregulation of ubiquitin gene transcripts increases the association between ubiquitin and muscle proteins in humans and the hydrolytic activity of the 20S- and 26S-proteasome complexes, causing extensive degradation of ubiquitin binding proteins (42). Although UPP plays a key role in muscle atrophy, it cannot complete the entire hydrolysis process by itself because myosin and actin normally align tightly in sarcomeres.

During muscle disuse, calpain and caspase-3 are activated with increased expression in the diaphragm and limb muscles (43). These two proteolytic enzymes can decompose proteins from the cytoskeleton, resulting in the release of actin and myosin from the sarcomeres. The above process contributes to the ubiquitination of myocyte proteins by a muscle-specific E3 ubiquitin ligase, allowing their degradation by UPP. Atrogin1 and MuRF1 are two key E3 ubiquitin ligases, the increase of which can be detected in the diaphragm and limb muscles during muscle atrophy (including disuse) (42). However, the role of calpain and caspase-3 in muscle atrophy remains controversial, and whether they play indispensable roles in ICU-AW requires further exploration (44).

### Autophagy–Lysosome System

The autophagy–lysosome system is an important pathway for disuse muscle atrophy in ICU patients (45) and is capable of degrading mitochondria or larger cellular structures. Vanhorebeek et al. found that uncontrolled autophagy could induce muscle atrophy in ICU patients and CIM animal models (46). Autophagy is a basic pathway of skeletal muscle catabolism and is upregulated under fasting, oxidative stress, and denervation conditions, leading to muscle protein degradation (47). Lysosomal degradation contributes to protein breakdown in denervated muscles, and the lysosomal protease (cathepsin-L) is significantly upregulated under atrophic conditions (48). Autophagy removes intracellular damaged and dysfunctional proteins and organelles and thus plays a vital role in cell homeostasis and skeletal muscle degradation. Autophagy is regulated by LC3, Atg7, the Forkhead box O proteins (FoxOs), and mTOR (49). siRNA knockdown of LC3 can partially prevent atrophy (45). Ablation of Atg7 leads to sarcomere disorder and muscle fiber denaturation, and Atg7-knockout mice exhibit muscle atrophy and weakness (50). Both the autophagy–lysosome system and the UPP regulate transcription through expression of the FoxOs, regulating proteasome-mediated protein breakdown and autophagy-mediated organelle clearance (51). FoxO1 deficiency is associated with a partial decrease in the expression of MAFbx, MuRF1, and the lysosomal enzyme cathepsin-L. The autophagy–lysosome system is dysregulated, leading to excessive degradation of muscle proteins, thereby aggravating muscle atrophy in patients with ICU-AW due to their characteristic long-time mobility restriction.

### Mitochondrial Damage

Mitochondria are important organelles involved in energy production, signaling, cell differentiation, and apoptosis. Under the state of aging and disuse, DNA mutations in the mitochondria lead to excessive ROS, destroying large molecules and impairing cellular and tissue function (52). The changes in muscle cell mitochondrial ultrastructure and the impaired function of the electron transport chain simultaneously occur in patients with acute critical illnesses. Damaged mitochondria produce ROS to induce proteolysis (39).

Corpeno Kalamgi et al. found that regulation of the mitochondrial size in ICU animal models might play a key role in CIM (53). The sizes and morphology of mitochondria are determined by the balance between mitochondrial fission and fusion. Mitochondrial fission is accompanied by DNA replication and autophagy, breaking down dysfunctional or damaged mitochondria, while damaged mitochondria can acquire the necessary genetic material and maintain normal functions through fusion (54). Specific loss of the fusion proteins Mfn1 and Mfn2 in muscles causes muscle atrophy and promotes overexpression of the cleavage proteins Drp1 and Fis1, enhancing fission and aggravating autophagy and muscle atrophy (55).

### Peripheral Neuropathies

Inflammation causes vascular endothelial cell activation and capillary leakage, causing effusion of toxic substances, leading to nerve end edema, cell damage, and axon degeneration (56). Muscle mass is affected by peripheral denervation, leading to an increase in mitochondrial fission and ROS, followed by activation of the proteasome and autophagy–lysosome pathways to degrade proteins (57). Recently, Nardelli et al. found that lowering motor neuron excitability reduced motor units and induced ICU-AW in septic model rats (58), whereas a 5-hydroxytryptamine 2C agonist (Lorcaserin) increased neuron excitability in rats and greatly improved their muscle strength (59). Peripheral neuropathy, a diabetic complication, can cause secondary muscle atrophy with activation of the glucose bypass metabolism–polyol pathway. In this pathway, large amounts of reduced coenzyme II (NADPH) are consumed, resulting in a decrease in NO synthesis or the glutathione (GSH) content, leading to a decrease in blood flow and production of a large quantity of free radicals causing nerve damage (60). Hyperglycemia also causes significant accumulation of advanced glycation end products (AGEs) and damages tissues through oxygen radical groups. Protein glycosylation in the nervous tissue causes a disorder in retrograde transport of the axon, interferes with protein synthesis in nerve cells, and leads to axon degeneration and atrophy. Eventually, the structure and function of nerve cells change, leading to a nerve conduction disorder.

### NMJ Lesions

The NMJ acts as a “bridge” between motor neuron terminals and skeletal muscles, and its integrity is essential for homeostasis of motor nerves and muscle fibers. Hypofunction caused by structural remodeling of the NMJ is an important cause of aging-related muscle atrophy (61). With advancing age, the structural stability of the NMJ decreases significantly, and degenerative changes occur in presynaptic motor nerve terminals and postsynaptic motor endplates (62). Through immunofluorescence staining, Gregorio Valdez et al. observed that some acetylcholine receptor (AChR) sites were unoccupied in aged mice, the motor endplates lost innervation, the axons of the nerve endings were swollen and deformed, and the postsynaptic membrane receptors were darkened and fragmented in the visual field. However, the integrity of the NMJ structure could be considerably improved through exercise and calorie restriction (63). In addition to aging, the NMJ morphology is affected by neuromuscular diseases. The NMJ is associated with the pathogenesis, diagnosis, and treatment of muscle atrophy (64). The ultrastructural changes in patients with spinal and bulbar muscular atrophy are specific, showing NMJ fragments with smaller motor endplates and enlarged synaptic gaps (65). Coincidentally, similar changes were identified in the NMJs of patients with muscular dystrophy (66).

**Figure 2:**
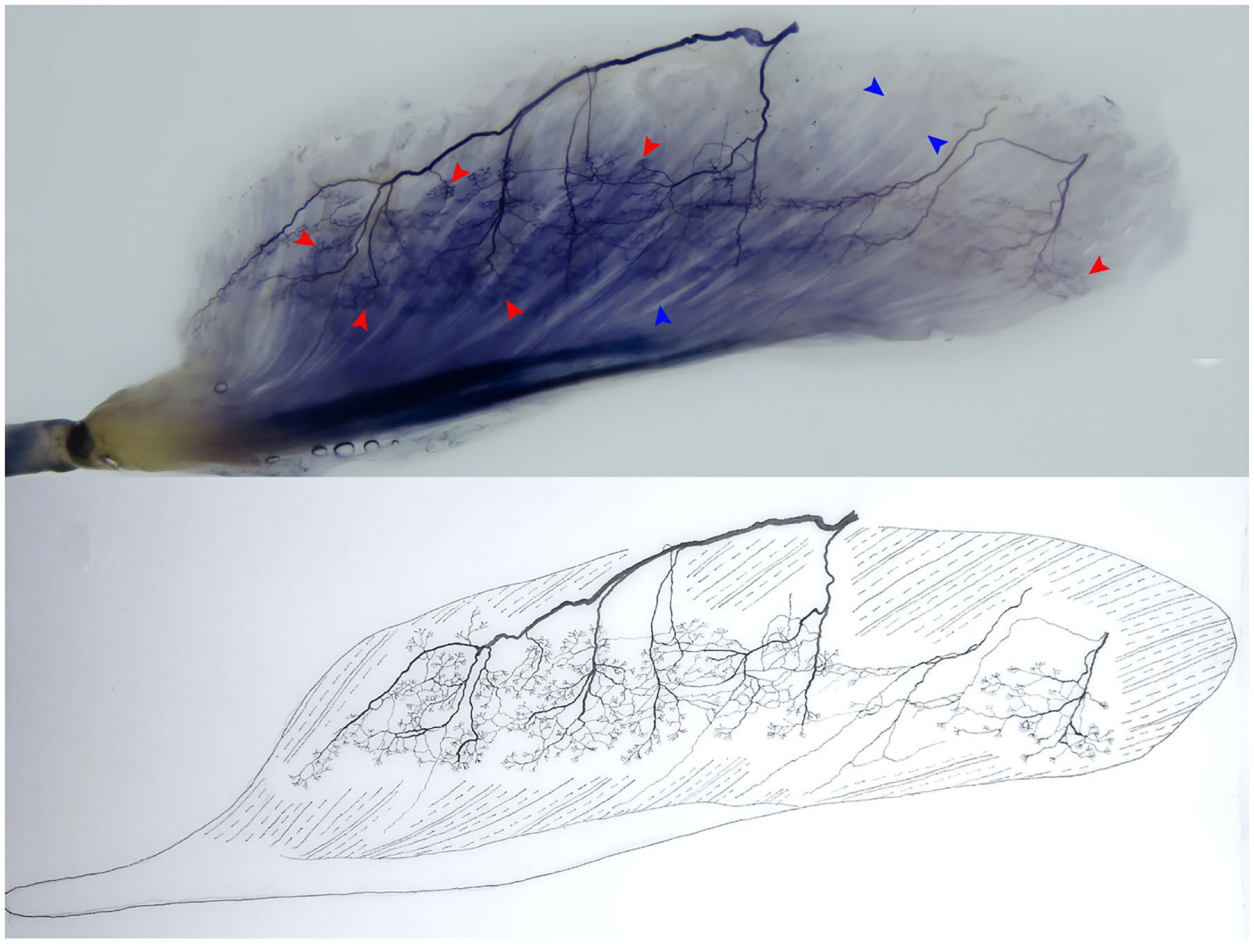
Modified Sihler's staining technique. The technique allows a clear three-dimensional visualization of terminal nerves (i.e., the site where an α-motor nerve ending attaches to the muscular fiber, namely, the motor end-plate), which cannot be observed by the naked eye. The relationship between the intramuscular terminal nerves and the muscle fibers (the NMJ) in the flexor hallucis longus is shown. The red arrowhead shows the terminal nerve, and the blue arrowhead shows the muscle fiber (right, superficial).

The association between muscle atrophy symptoms in patients with ICU-AW and structural changes in the NMJ is unclear. The NMJ may be an important direction for future research on the mechanisms and treatments of this disease. Therefore, the morphological changes in the NMJ must be confirmed in the ICU-AW animal model in early-stage studies. At present, in addition to the aforementioned immunofluorescence method, our previous studies have proven that the modified Sihler's staining technique can be used to observe the relationship between intramuscular terminal nerves and muscle fibers (the NMJ as a whole) (67, 68). This technique is the best way to track nerve terminals in muscles without damaging skeletal muscle integrity (Figure 2) and to clearly reveal intramuscular nerve terminal aggregation areas three-dimensionally, invisible to the naked eye. Future studies should confirm the morphological changes of the NMJ from both macroscopic and microscopic perspectives.

The C-terminal agrin fragment (CAF), a soluble fragment, remains in tissues after local cleavage of agrin by neurotrypsin during NMJ remodeling. Excessive cleavage of agrin leads to functional disintegration of the NMJ and triggers continuous muscle atrophy (69); thus, a large amount of CAF enters the blood circulation. Circulating CAF may function as a biomarker of muscle atrophy (70); it is detectable in human serum and can be quantified using enzyme-linked immunosorbent assays. Muscle atrophy should be suspected when elevated CAF concentrations are detected in the blood (71). However, current research on CAF is mostly focused on age-related muscle atrophy, with small sample sizes (72). The change in serum CAF levels in patients with ICU-AW before or after disease onset and its utility as an early biomarker need to be further verified.

Discussion of the above mechanisms focuses on the acute phase of critical illnesses and short-term animal models. Although muscle atrophy persists for several months in patients with ICU-AW after hospital discharge, studies on potential molecular mechanisms are limited (73). Sustained proteolysis is not observed, although muscle regeneration is impaired (74). The phenomenon of muscle atrophy after discharge requires attention for a complete understanding of ICU-AW. However, a thorough understanding of the mechanism of the second part of the ICU-AW process requires extensive follow-ups, reviews, and intensive research.

## Current Diagnosis and Intervention Procedures

### Diagnosis

Regrettably, a real “gold standard” diagnostic test for ICU-AW has not been identified even in the last Clinical Practical Guideline (75). ICU-AW can be diagnosed in three ways: manual muscle testing, electrophysiology (EMG and nerve conduction studies), and muscle or nerve tissue pathology. Manual muscle testing refers to dividing the strength of functional muscle groups of the limbs into grades of 0–5 according to the MRC scoring (i.e., none, weak, poor, acceptable, good, and normal). The scores obtained from the preselected muscle groups are summed to obtain a total score use to evaluate the overall motor function. If the evaluation is performed in six bilateral muscle groups (wrist flexion, forearm flexion, shoulder abduction, ankle dorsiflexion, knee extension, and hip flexion), the total score ranges from 0 (complete paralysis) to 60 (normal muscle strength) (75). The MRC score is highly reliable in patients with Guillain–Barre syndrome, with successful implementation in critically ill patients (76). In a few prospective studies of mechanically ventilated patients, a total MRC score of 48 was defined as a threshold value for “ICU-acquired paralysis” (77). Although manual muscle testing is simple and easy to perform, ~25–29% of ICU patients are conscious during testing (4). Second, electrophysiological changes in the muscles can be detected 24–48 h after ICU-AW onset and appear before clinical symptoms (78). EMG is important to identify the underlying pathology causing weakness (CIP, CIM, CIPM). However, electrophysiological testing is not routinely used in clinical practice. Third, nerve and muscle biopsies can provide critical information, but biopsy is invasive and may cause complications in routine clinical use; hence, it is no longer advised, except in the context of scientific research.

### Nutrition and Supportive Therapy

Since the mechanism of ICU-AW is unclear, no specific drug or effective treatment is currently available. However, appropriate interventions can improve the prognoses of patients to some extent. Muscle biopsies of critically ill patients showed lower glutamine and protein/DNA levels and a higher extracellular water concentration (79). Low endogenous glutamine levels are insufficient to meet the needs of the body. Novak et al. found that parenteral glutamine supplementation could reduce the mortality in ICU patients (80). Furthermore, arginine is used for maintaining protein homeostasis and nutrition in patients with burns or sepsis.

Antioxidant therapy, another nutritional regimen, is beneficial for patients with ICU-AW. Excessive free radicals and reduced endogenous antioxidant mechanisms are the recognized causes of MOF and septic shock. The concentration and absolute synthesis rate of whole blood GSH are decreased in patients with burns (81). Muscle biopsies in critically ill patients also show decreased GSH levels, and its supplementation can reduce the oxidative stress index. The effect of the GSH precursor N-acetylcysteine (NAC) administration is more pronounced as it can scavenge oxygen free radicals and increase the GSH reserve (82).

Hormone therapy maintains a key position in treatment of muscle atrophy. Growth hormone (GH) secretion increases during acute critical illness, and secondary reduction of insulin-like growth factors I (IGF-I) and II (IGF-II) causes a negative nitrogen balance (83). High doses of recombinant human GH can improve the nitrogen balance and peripheral muscle strength. However, it has side effects such as hyperglycemia, increased visceral oxygen consumption, and increased mortality and MOF rates (84), which indicate that blocking skeletal muscle catabolism during the acute phase of critical illness may not be the best option. Therefore, further research is required to determine the effectiveness of this treatment.

## Summary and Prospects

Although ICU-AW is a common finding in critical care, our understanding of this disease is limited, and many associated problems require solutions. [1] The bridge between nerves and muscles, the “NMJ”, has not received sufficient attention, and few studies have been conducted in this regard. [2] The role of mitochondria in ICU-AW is unclear. [3] The balance between protein degradation and synthesis at each stage of disease development is unknown. [4] The use of CAF as a biomarker for early diagnosis of ICU-AW is unclear. [5] The effective use of NMBAs and dosage should be determined. Future research should focus on the pathophysiological mechanisms of ICU-AW, as development of an acceptable treatment strategy requires clear understanding of the etiology for patients to adapt to the society and improve their quality of life.

## Author Contributions

WW, CX, XM, XZ, and PX performed the literature search, wrote the first draft of the manuscript, and which was critically reviewed by PX. All authors contributed to the article and approved the submitted version.

## Conflict of Interest

The authors declare that the research was conducted in the absence of any commercial or financial relationships that could be construed as a potential conflict of interest.
